# Sources of Occupational Stress among Office Workers—A Focus Group Study

**DOI:** 10.3390/ijerph19031075

**Published:** 2022-01-19

**Authors:** Larissa Bolliger, Junoš Lukan, Elena Colman, Leen Boersma, Mitja Luštrek, Dirk De Bacquer, Els Clays

**Affiliations:** 1 Department of Public Health and Primary Care, Ghent University, 9000 Ghent, Belgium; colman.elena@outlook.com (E.C.); leen.boersma8@gmail.com (L.B.); dirk.debacquer@ugent.be (D.D.B.); els.clays@ugent.be (E.C.); 2 Department of Intelligent Systems, Jožef Stefan Institute, Jožef Stefan International Postgraduate School, 1000 Ljubljana, Slovenia; junos.lukan@ijs.si (J.L.); mitja.lustrek@ijs.si (M.L.)

**Keywords:** occupational stress, office workers, qualitative research, phenomenology, focus groups

## Abstract

Workplace stress remains a major interest of occupational health research, usually based on theoretical models and quantitative large-scale studies. Office workers are especially exposed to stressors such as high workload and time pressure. The aim of this qualitative research was to follow a phenomenological approach to identify work stressors as they are perceived by office workers. Six focus groups with office workers of different occupations were conducted in Belgium and Slovenia. A total of 39 participants were included in the study. We used the RQDA software for data processing and analysis and the seven job-quality indices of the European Working Conditions Survey (EWCS) to structure our findings. The results show that work intensity and social environment proved to be main stress categories, followed by skills and discretion, prospects, and working time quality. The physical environment and earnings were not covered in our results. We created organisational (structural/process-oriented and financial) stressors and office workers’ physical health as two additional categories since these topics did not fit into the EWCS. While our findings mainly confirm data from existing occupational stress literature and emphasise the multi-level complexity of work stress experiences, this paper suggests that there are relevant stressors experienced by office workers beyond existing quantitative frameworks.

## 1. Introduction

Based on a systematic literature review, annual costs of occupational stress in Europe vary across countries from, e.g., USD 703.12 million in Sweden, USD 2.27 billion in Denmark, USD 3.33 billion in Switzerland, USD 4.36 billion in France, to USD 5.42 billion in the United Kingdom. Within the EU-15, occupational stress costs society USD 26.47 billion per year [1]. A joint report from the European Foundation for the Improvement of Living and Working Conditions and the European Agency for Safety and Health at Work showed that 25% of workers reported experiencing work-related stress for most or all their working time, while work intensity was mentioned as the key stress factor. Nearly 80% of managers stated that they experience occupational stress and more than half considered time pressure as their main concern. Approximately one out of four workers reported adverse health effects caused by their work [2].

The consequences of such occupational stress include a variety of adverse outcomes for employees and organisations. It can lead to absenteeism, poor time-keeping, high staff turnover, or even aggressive communication or bullying. The workers’ performance can be negatively influenced, leading to errors or poor decision making. Further outcomes are of psychosocial and health-related nature, such as anxiety or depression, burnout, problems in work or private relationships, sleeping disorders, musculoskeletal pain, or cardiovascular problems [3].

Office workers are additionally exposed to specific stressors such as increasing amount of demanding knowledge work requiring complex formal training, expected high productivity and creativity, and the demand to work flexible hours and in changing places and environments [4]. Moreover, non-stop high mental workload and ongoing technological development, requiring talent to adapt to such changes and continuous learning, increases stress among office workers [5].

Previous qualitative research aimed to categorise self-perceived causes of stress at work. A study of Bhui et al. [6] provides a good overview about occupational stress categories based on both office-based and other workers. These categories include working conditions, the nature of the job, management practices, life events, and financial factors. In focus group studies aiming to gain insights into experiences of occupational stress by university staff, Gillespie et al. [7] and Bos et al. [8] described several stressors, including job insecurity, work overload, lack of time for research, frequent interruptions during work, and a lack of promotion prospects as major causes of stress.

However, such focus group studies focusing on causes of occupational stress are scarce. A high number of focus group studies in the field of occupational health investigate consequences of work stress such as burnout [9,10], frequent sickness absence [11], general health at work [12], psychosocial risks at work [13], or patient safety as a consequence of stress among nurses [14]. Additionally, the study population of the majority of such studies is difficult to compare to office workers, since they often include healthcare professionals such as medical doctors or nurses, which are exposed to different work settings, including patient care [9,10,14,15,16,17].

Psychosocial work stress is usually described with the help of theoretical models such as the Job Demand-Control model [18] and the Effort-Reward Imbalance model [19]. To empirically test these models, quantitative data are often collected within large-scale studies. Quantitative research traditionally offers large data sets and statistically powerful methods to analyse associations between a limited number of predefined work stressors and outcomes. However, stress is a complex and multivariate process, and stress experiences are based on self-perception and therefore highly subjective [20]. A quantitative approach has major shortcomings when attempting to fully capture stress in its complexity and subjective nature, since stress is typically assessed using closed-ended questions, which does not allow clarifying follow-up questions and does not initiate discussions.

While subjectivity is often perceived as a drawback in quantitative research, qualitative methodologies such as phenomenology embrace the opportunity to put oneself in the shoes of study participants in a face-to-face manner [21]. This study takes advantage of a qualitative phenomenological research approach. Through open, engaging, and inclusive discussions during focus groups with office workers, a variety of perspectives and a diversity of work stressors could be captured. This enabled us to conduct an in-depth analysis of work experiences, leading to a better understanding of the workplace contexts in which occupational stressors are manifested. Additionally, through clear and structured data collection and analysis approach, our qualitative research on work stress aims to contribute to a deeper understanding of existing theories and quantitative data. Furthermore, by connecting our qualitative study to a theoretical framework based on quantitative research, we strengthened the quality of our research output [22].

The aim of this research was to identify work stressors as they are perceived by office workers in two different countries, using the 6th European Working Conditions Survey (EWCS) [23] as a theoretical and structural framework to present our findings.

## 2. Materials and Methods

This paper follows the Standards for Reporting Qualitative Research (SRQR) [24].

### 2.1. Qualitative Approach

Phenomenology investigates people’s lived experiences and has become a major research method in a variety of research disciplines, including psychology and human sciences. One example is a study which focuses on the experienced thoughts, feelings, and worries of family members waiting for a loved one undergoing a major surgery [25].

We applied such a descriptive phenomenological approach in our focus groups. Kitzinger [26] described focus groups as discussions in groups, which are organised to explore a specific set of topics or problems. Such focus groups have several benefits compared to other qualitative research methods. First, focus groups stimulate discussions among the participants about their own experiences, through which true feelings and beliefs are revealed due to the interaction and social gathering. Second, through focus groups, a multiplicity of views and emotional processes can be captured, which are particularly useful in research on complex social phenomena. Third, researchers have the chance to gather a larger amount of information in a shorter amount of time, compared to individual interviews [27].

Descriptive phenomenology looks at pure experiences of people, aiming to remain close to their statements given, without interpretations of the investigating researchers [28]. Through our focus groups, we explored the perception of stressful work experiences of our participants in their work environment.

### 2.2. Sampling Strategy and Context

The participants were recruited via the researchers’ professional and personal network and initially contacted with a recruitment letter shared either personally or via email. In Belgium, seven institutions were contacted, four of which did not participate due to limited time resources, while three agreed to participate. In Slovenia, three institutions were contacted, all of which agreed to participate.

Studies focusing on the perception of employers and management show that those higher ranked tend to report differently than their subordinated office workers on what causes occupational stress and how it is experienced within their organization [7,9,11,17]. In that regard, attention was paid to avoid hierarchical discrepancies between the individual focus group participants. By including office workers without their superiors, an environment for open and honest communication and exchange of experiences could be created.

This qualitative study is part of the STRAW (STRess At Work) Project based in Belgium and Slovenia, researching stress in academia [29]. Therefore, the focus groups were conducted in Ghent and Brussels, Belgium, and in Ljubljana, Slovenia. Per country, one focus group was organised among co-workers of the main researchers within academic working environments. The remaining four focus groups took place with office workers employed in an architects’ office, an international non-governmental organisation, a publishing house, and a statistical office. This sampling strategy allowed a representation of a diverse set of office occupations from the two countries. With this heterogeneity and number of participants, we judged that we reached sampling saturation, and we stopped the recruitment. The focus groups were held during lunch break in meeting rooms of the participants’ workspaces. Sandwiches and beverages were provided as an incentive and small reimbursement and in order to create a relaxed atmosphere.

### 2.3. Ethical Clearance

This study received ethical clearance from the Commission of Medical Ethics of the Ghent University Hospital, Belgium (No. 2018/1058, dated on 25 September 2018), and the Ethics Committee of the Faculty of Arts at the University of Ljubljana, Slovenia (dated on 24 July 2018). All participants received an information letter and signed an informed consent before their focus group participation.

### 2.4. Data Collection Methods and Instruments

The focus groups took place between October 2018 and January 2019. The focus groups in Belgium were conducted in English (since the first author and several participants were non-native Dutch speakers, English was considered as the most inclusive language), and the focus groups in Slovenia were conducted in Slovenian. 

Each focus group lasted approximately 90 min without any breaks. Participants completed a survey about their sociodemographic information before the focus group started. During each focus group, one main researcher was present as the moderator, who was responsible for asking the questions and guiding the discussion. A second researcher was responsible for taking notes, the audio recording, the time management, and double checking at the end of the focus group if all questions were discussed. The focus groups were recorded with several laptops or smartphones spread across the room to assure that all statements could be captured.

The moderators followed a predefined semi-structured focus group guide, including a set of open-ended questions, focusing on the participants’ experiences of what causes their work stress. The focus group guide was developed based on the guidelines on how to organise and run focus groups as part of the Management Standards for Tracking Work-Related Stress by the UK Health and Safety Executive [30]. The focus group guide remained the same throughout all six groups and is available in the Appendix A. 

### 2.5. Data Processing, Analysis, and Framework

The audio recordings were fully transcribed and pseudonymised by using the participants’ initials. We used the R package for Qualitative Data Analysis (RQDA) [31] to process the transcripts and to create an initial overview by using the RQDA functions of colour coding quotes into topics and creating matrices of related topics to facilitate first result discussions. We followed an inductive/theory-building approach by highlighting quotes indicating any sort of stress sources mentioned by the participants. We processed each transcript several times in order to prevent missing any quotes. We grouped these quotes independently from each other into self-developed RQDA categories (e.g., time pressure).

As a next step, we merged the English and Slovenian quotes into one data set and discussed each category, making sure overlapping topics were named with a representative and precise category name. During this phase, the Slovenian quotes were translated into English. We picked an exemplar quote per category, choosing them by how well they presented the category and by how self-explanatory they were to readers who were not present during the focus groups. During this process, only the focus group numbers 1 to 6 were added to the exemplar quote, without any information on the individual participant for de-identification purposes.

The categories were then compared to and structured by the three levels of the EWCS [23], namely, sub-indicators, indicators, and job-quality indices. For example, the quote representing the category time pressure was categorised as working at a very high speed (sub-indicator), quantitative demands (indicator), and work intensity (job-quality index). To enhance validity of this data analysis approach, investigator triangulation [32] was used, meaning we involved all project researchers in discussions to harmonise different perspectives and to structure our results using the EWCS [23].

The EWCS [23] is one of the broadest and most widely known job-quality frameworks based on a repeated study, aiming to contribute to European job quality improvement policies. It includes seven job quality indices: physical environment, work intensity, working time quality, social environment, skills and discretion, prospects, and earnings. This study made use of the sixth version of the EWCS [23], implemented in approximately 43,000 face-to-face interviews from workers in a variety of sectors such as construction, agriculture, services, and public administration across 35 European countries in 2015. It consists of a basic survey and structured interviews with three open-ended questions [23].

## 3. Results

In total, six focus groups, consisting of 5 to 10 participants, were conducted with a total of 39 participants, 18 in Belgium and 21 in Slovenia. Of the participants, 24 were female and 15 were male, with their ages ranging from 23 to 56 years (mean 35 years). Most participants (35) were working full-time with an employee tenure for their current employer between 2 weeks and 20 years. Moreover, 1 participant indicated their highest level of education as vocational education, 11 held a bachelor’s degree, 22 a master’s degree, and 4 a PhD degree or higher, while 1 indicated other educational level. Participants of focus groups 1 and 4 worked in academic settings (15 participants), while focus groups 2, 3, 5, and 6 were recruited from non-academic settings (24 participants).

The following results are structured by the job quality indices and their indicators of the EWCS [23] (Table 1) and outlined with exemplar quotes, while indicating from which focus group (FG1–FG6) they are cited.

### 3.1. Physical Environment

The physical environment index was not discussed in any of the focus groups.

### 3.2. Work Intensity

#### 3.2.1. Quantitative Demands

As expected, working at a very high speed could create a feeling of being in a hurry and general time pressure. Tight deadlines for tasks on short notice or a final phase of a project were mentioned as additional stressors. FG3 mentioned that during the year, strict deadlines occurred, such as Christmas or summer holidays, or research needed to be published as stated by FG1. Knowing that you do not have the capacity or enough time to get the job done, as explained by FG1, was indicated by several participants as a major stressor. Constant availability during working hours and having a feeling of there being no work limit were other topics mentioned. Several FGs indicated that working while being frequently interrupted by colleagues or messages was frustrating. FG1 added that small interruptions could cause the unintentional procrastination of major tasks. FG2 described that disturbances by colleagues could lead to loss of focus and a strained work environment. Furthermore, a lack of good time management was described as highly stress-inducing. Participants found it stressful to be on time for each meeting, to find time to supervise subordinates, to set priorities, or to correctly estimate the time needed for different tasks. Statements of several FGs overlapped when describing the need for multitasking while handling several high-priority tasks at once as a source of stress. Multiple participants stated that a high or fluctuating workload could make the workplace very hectic. FG6 mentioned that scheduling tasks could be problematic due to the extensive workload:

*“Yes, I would say time pressure, well, but not in the sense, I don’t know, that I couldn’t schedule it properly, but the thing, I don’t know, the tasks just keep coming additionally, right, additional tasks, right. So yes, too extensive workload.” (FG6)*

#### 3.2.2. Pace Determinants and Interdependency

Several participants described work pace set by others as a source of stress. Being dependent on others’ work or the pressure put by colleagues or clients on them to finish jobs at an unreasonable speed was described as frustrating. FG2 mentioned that pressure exerted by colleagues could be particularly stress inducing. FG3 explained that clients often have high or unrealistic expectations of employees to finish their requests or to do a good job since they are not aware of their overall workload:

*“And that the client see that as things always that you are only working on his project and that they don’t realize that you don’t have only one project, you have multiple. And they think you are, they are the only one who you have to think about, which is not true of course. Eh, but it’s also what the client, I think it’s also part of what the client expects. Because they, they go to this big office and they see a capacity so it should be fine. You cannot really tell them or explain that to them because they’ll not understand that is. But you have so many people, how can that be?! That’s … that’s indeed it’s a stressful situation.” (FG3)*

#### 3.2.3. Emotional Demands

Feeling alone in a project or in the organisation and experiencing a lack of meaning of their work was described as demanding by FG2 and FG5. Furthermore, insecurities about their own abilities to perform the job, self-doubt, and fear of failure were further mentioned as stressors. Related to this were topics such as wanting to do a good job and high self-expectations. Additionally, the intention to help colleagues was mentioned by FG2. Frustrations caused by feeling unfairly treated when compared to others was mentioned by several participants. FG2 and FG3 stated that having to handle unsatisfied clients and having unfriendly work contacts could increase the emotional demands of their job:

*“So I have no [laughs], … not many friendly contact.” (FG2)*

### 3.3. Working Time Quality

#### 3.3.1. Duration

FG2 mentioned commuting to and from work as a relevant stressor, since it could extend the work-related time investments or could cause a delay in arriving on time:

*“… but then there was traffic … [laughs] so was a little stress.” (FG2)*

#### 3.3.2. Flexibility

FG3 stated that having to work overtime during evenings and weekends to meet the work demands could be stressful. FG1 mentioned difficulties to maintain a healthy work-life balance:

*“The work-* *life balance is for me very difficult … I want still to do something for work because then it’s finished but if I do that then I feel regret that I don’t take private time but if I do my private time then I regret that I was not working.” (FG1)*

### 3.4. Social Environment

#### 3.4.1. Adverse Social Behaviour

Interpersonal conflicts were mentioned by several participants, for example, when colleagues do not get along. Even bullying was mentioned. FG6 explained that colleagues meddling in private affaires or an adverse attitude of colleagues, such as someone who has the knowledge to help but will not do so, were perceived as strong stressors. Additionally, FG2 and FG3 viewed working with people for the first time or with people who are overwhelmed by their workload as a source of stress. FG2 specified that their stress and their inability to cope with it could result in crossover stress. The uncertainty caused by comparing oneself to colleagues or competing with them was considered to create doubts about their own work performance. FG2 and FG3 added that mistrust or dishonesty among colleagues could have a negative impact on their teamwork. FG3 felt that lack of transparent communication could cause unnecessary tension and stress:

*“Lots of things could have been avoided by different communication … it’s the way how you communicate with people. If it’s an email, if it’s a very different approach when you just have somebody in a meeting and explaining to them … that’s a very different approach which creates stress or tension that’s not really necessary. So that’s a bit a big thing just to have open communication about other things instead of just doing it through email or … keeping it to yourself.” (FG3)*

#### 3.4.2. Social Support

A relevant factor mentioned by FG2 was the peer pressure when starting a new job to perform at the same level as others. Several participants revealed that not being able to find colleagues who are qualified to help could cause stress. Furthermore, the inability to rely on colleagues’ assistance or having to do the tasks for which someone else is responsible could cause tension, as explained by FG3:

*“Then you experienced that she, eh, doesn’t really want to do that and she actually doesn’t do it, not at all. So after three weeks I think I had some, five adjusted problems. And, yeah, each three, two or three days when I was at the office and I checked, she really didn’t want to do it. And, eh, after three weeks, we decided that, yeah, she really didn’t want to do anything for me… so, that was really strange, it was the first time that actually someone came in and that, yeah, didn’t really want to help and didn’t want to cooperate in, in the team. That was really strange.” (FG3)*

#### 3.4.3. Management Quality

Superiors avoiding making decisions or taking responsibility and therefore taking a wrong direction, making no improvement despite several complaints directed at leadership causing unfulfilled expectations of subordinates, as well as unhelpful and unsupportive leadership were mentioned as stressors. Some participants stated that poor work organisation from superiors was a negative example and caused tension within the team. FG1 described a lack of feedback from superiors, and therefore uncertainty if their own work is being done as expected, as stressful. FG5 explained how authoritarian leadership could undermine work quality. Moreover, FG3 mentioned a lack of acknowledgment and appreciation from superiors as a major stressor:

*“It is, let’s say, something to work on for the management here. Because they, it’s, I, I only once have experienced that somebody said to me ‘you have done a good work’.” (FG3)*

### 3.5. Skills and Discretion

#### 3.5.1. Cognitive Dimension

FG3 mentioned tackling new and unknown tasks as major stressors. FG1 mentioned that facing challenges, unforeseen tasks, or unanticipated events, which could not be planned or which required a change of plans, were all stressful:

*“Quite often you have a schedule in your mind like, ok, by then I need to do that, by then that and quite often with all of this just pop up like, yeah, can you still do that and quite big challenges not just small ones. So it’s often quite difficult to manage deadlines because there are a lot of unforeseen challenges.” (FG1)*

#### 3.5.2. Decision Latitude

FG2 stated that the need to work independently as expected by a superior could be stressful:

*“We have a lot of liberty … freedom in our job and it’s very important… for me important. But I know that this can give stress for people who just don’t have the limits and can go a little bit in all the ways they want. So that’s maybe something happens a lot here.” (FG2)*

#### 3.5.3. Organisational Participation

FG5 mentioned that a lack of being included in improving work organisation or processes and not having the chance to influence decisions about their own work were relevant stressors:

*“We feel that if something were to change here and if there would be more dialogue, that is, perhaps … what is wrong, more dialogue with the people who work in individual areas, because they are the ones with the best solutions, let’s say, right. And it happens often that they are instead thinking about these solutions in a top-down manner, instead of thinking from the point where people know, in this case they would simply get to solutions faster, right. And that’s for every area and that is then, hmm… that is, for, for those people, that aren’t us perhaps … we do talk quite a bit … but some people know how they could organize things, but they cannot speak out at all because, well, they are simply afraid.” (FG5)*

#### 3.5.4. Training

FG2 mentioned that stress could be induced by a lack of training when starting a new job:

*“What I could say is one thing that I could say stressful is that the person who’s supposed to give the handover and to coach me, train me, is sick for the moment. So not really easy to start that way so I need to pick up information where I can.” (FG2)*

### 3.6. Prospects

#### 3.6.1. Employment Status

A short-term or non-permanent employment contract and changes in a contract were described as challenging by FG2 because of the insecurities they could cause:

*“So not everyone have long-term contracts. They have short contracts and sometimes we don’t have the answer enough on time. And so I think it’s really stressful for the people because they don’t know if they will stay or not. I had experienced to someone who didn’t know one week before the end of his contract. That’s something, sometimes we cannot do or we don’t know how to change because, yeah, it’s budget and organization.” (FG2)*

#### 3.6.2. Career Prospects

Several participants mentioned increased responsibility due to career advancement as a stressor. Moreover, it was explained that making a long-term career-focused decision was highly stressful because of the impact on their career and overall life. A lack of vision or unrealistic long-term goals by the leadership could cause discomfort, as explained by FG4. Additionally, FG1 mentioned rejection of funding applications was stressful:

*“Applying for project and having a lot of negative, failure projects. So something you submit and you get it back, they don’t accept.” (FG1)*

#### 3.6.3. Job Security

FG2 mentioned that overall uncertainty about the future of the job led to insecurities:

*“What I also see a lot is that with the restructuration. And the information, they hear something but they don’t know what really gonna happen and the people on top they can’t tell, they have to lie sometimes. It gives a lot of stress to the people because ‘Next week, am I gonna work here? Is this shop gonna be open? Are we gonna move to there?’ And the people on top don’t know yet. And that gives everywhere you feel the same stress because of the uncertainty they heard already.” (FG2)*

#### 3.6.4. Downsizing

Frequent changes in the team, such as colleagues leaving or starting, was mentioned by several participants to have a strong impact on their team. FG5 expressed general concern for downsizing in the future due to the aging of their team. FG1 and FG3 mentioned that they found it difficult to work when the number of employees at the workplace has been reduced over the years. They explained that it could increase their workload when there is a lack of (qualified) staff or a high turnover in staff:

*“The main problem in our department is the lack of staff for the number of project that there are… when I started to work here 14.5 years before, I think that were 5 people who have been retired or out of contract who haven’t been replaced. And the staff was about 55, maximum 60 persons and now we are almost doubled with a lot less [admin] staff.” (FG1)*

### 3.7. Earnings

The earnings index was not discussed in any of the focus groups.

After comparing to and structuring our categories by the EWCS [23], a number of quotes did not fit any job quality indices. We discussed these quotes and their categories and developed two additional job quality indices, including sources of work stress which are not described in the EWCS [23].

### 3.8. Organisational

#### 3.8.1. Structural and Process-Oriented

Days packed with meetings or with presentations were stressful for several FGs. FG1 mentioned their irritation with inefficient structures and procedures within the workplace and with having to make decisions while considering the multiple stakeholders involved in a project. FG3 explained that the first phase of a project could be stressful because of finding the appropriate working rhythm. A lack of equipment, tools, or other appropriate infrastructure was described as stressful. Furthermore, practical and technical issues such as IT problems were stated by several participants. FG2 and FG3 added that a lack of organisational structure and structural changes in the organization could be confusing and stressful. FG4 and FG6 found it challenging not having control over general deadlines and lacking clear definitions regarding who is responsible for which task. Additionally, FG4 found unstructured tasks, tasks without any clear definition, and tasks that were not a legitimate part of the job stressful:

*“The way I see this, what makes stress for me, like she mentioned, and then for example I am forced to go over this thing again and then I feel like stress, because I feel I need to do a thing again that doesn’t have a goal.” (FG4)*

#### 3.8.2. Financial

Financial pressure due to limited budgets or delayed refunds of expenses were described as worrisome for FG2:

*“There is also the money issue. Because they have to get the money back for everything they spend and it sometimes, it takes time.” (FG2)*

### 3.9. Physical Status

#### Employees’ Health

Topics such as being hungry during work and lack of access to high-quality food at the worksite were described as stressful for several FGs. Moreover, poor sleep quality or lack of sleep were mentioned as further stressors by FG4. Ill health and physical conditions were discussed by FG5:

*“And if you have a health condition, currently for half a year, my eyes are playing with me, I cannot read in the evenings, I cannot read over the weekends as much as I used to and that means … enormous quantity of books that lay there and I haven’t read them. And no one will instead of me.” (FG5)*

## 4. Discussion

While psychosocial work stress research is traditionally based on quantitative data sets, this study is based on focus groups with office workers, reflecting their views on the causes of work stress.

We made use of the EWCS [23] and its seven job quality indices to structure our results. Furthermore, we discuss our findings in connection with two of the most widely used occupational stress models, namely, the Job Demand-Control model [18] and the Effort-Reward Imbalance model [19].

The work intensity index focuses on the level of work demands. Depending on the source, between one-third [23] of workers and 80% of managers in Europe are experiencing work-related stress due to high work intensity and time pressure [1,2]. This was corroborated in our focus groups and in a similar focus group study with university staff in which quantitative demands as well as pace determinants and interdependency were frequently discussed sources of work stress, hindering positive discussions and social interactions with colleagues [8]. These results are in line with the demands subscale of the Job Demand-Control model [18] and the effort subscale of the Effort-Reward Imbalance model [19]. A highly relevant indicator of the EWCS [23] is emotional demands, which was also described by our participants as stress-inducing. The EWCS [23] pays more attention to it in comparison to other frameworks and models. Neither the Job Demand-Control model [18] nor the Effort-Reward Imbalance model [19] include emotional demands in their models or corresponding questionnaires, which shows a clear shortcoming, since emotional demands at work can cause adverse health consequences such as higher risk of long-term absence due to illness [33].

The working time quality index measures time-related aspects such as long work duration, atypical working time, working time arrangements, and flexibility. The general working time quality has improved since 2005 according to the EWCS [23], for example, with 43% of workers in Europe following a very regular working schedule. While long working hours due to commuting to and from work were described as stressful, atypical working times or working time arrangements were not mentioned by our participants, most likely due to their office environments and an absence of shift work. A total of 22% of workers in Europe have to work in their free time several times a month to fulfil their work demands [23]. This is in accordance with our results since working during spare time and thereby experiencing work-life conflicts was described in our focus groups as highly relevant. Positive aspects have been observed in other research, for example, the flexibility in academic jobs, but there is also the tendency to work extra hours, leading to a poorer work-life balance [34]. The Job Demand-Control model [18] does not include these topics, while the Effort-Reward Imbalance model [19] does consider the influence of work on private life with items such as *“Work rarely lets me go, it is still on my mind when I go to bed”*. However, the model categorises it as overcommitment, which conceptually differs from work-life balance. While our participants mentioned work-life conflicts as both a source and consequence of stress, many quantitative studies conceptualise it solely as a stress outcome. For example, a study among Australian academics showed that job stress is a clear predictor of work-life conflicts [35]. However, the Job Demand-Control model [18] and the Effort-Reward Imbalance model [19] lack a focus on work-life balance.

The social environment index includes adverse social behaviour such as harassment or bullying as well as positive and supportive social work relationships. According to the EWCS [23], approximately 16% of workers in Europe experience such adverse social behaviour. In our focus groups, it became evident that a lack of social support and adverse social behaviour from colleagues were major sources of work stress. As found by quantitative studies, interpersonal mistreatment or workplace incivility are often subtle and more common than physical violence or psychological aggression but have many consequences such as lower job satisfaction, impact on both individual and organizational performances, decrease in physical health, and a greater risk of leaving the job [36,37]. In contrast, positive social support and being able to rely on each other were shown to be protective for workers, potentially preventing burnout [9]. Management quality is a specific indicator, which also proved to be highly relevant in our focus groups since a lack of such quality was experienced as stressful for our participants. Another focus group study found that a lack of help and support from the manager and an unsupportive leadership increases negative emotions among workers and an emotional distance from the workplace. Furthermore, a lack in leadership results in a lower perceived responsibility to one’s own work and a lower barrier for calling in sick [11]. Both the Job Demand-Control model [18] and the Effort-Reward Imbalance model [19] focus on social interactions at work in their models and corresponding questionnaires. While Karasek et al. [18] set high value on the social support subscale of the Job Demand-Control model [18] and dedicate several items to positive social experiences with superiors and colleagues, Siegrist [19] additionally includes adverse experiences in the Effort-Reward Imbalance model [19] with items such as *“I am treated unfairly at work”* belonging to the rewards subscale.

The skills and discretion index assesses the organisational participation, learning and training opportunities at work, decision latitude, and cognitive dimension of a job. According to the EWCS [23], only 33% of subordinates are included in decision making, while 80% of managers in Europe are directly involved in decisions affecting their own work. This is in line with our results, since not having the chance to be involved in organisational discussions was described by our participants as stressful. A lack of suitable training when starting a new job was also identified as a strong stressor for our participants. Decision latitude was of high relevance in our focus groups and proved to be a complex topic. On the one hand, freedom, or the liberty to make one’s own decisions, was described by our participants as helpful. On the other hand, depending on the person and situation, it was described as very stressful when work limits were not set. A focus group study by Ironside et al. [9] confirmed that high responsibility can result in insecurity, self-doubt, or even burnout. However, the Job Demand-Control model [18], in which low control is associated with stress, measures skill discretion and decision authority with items such as *“I have a lot to say about what happens on my job”*, focusing solely on positive aspects of high decision latitude. Furthermore, the cognitive dimension such as handling unforeseen problems proved to be highly stressful for our participants. The Effort-Reward Imbalance model [19] looks into the cognitive dimension with items such as *“Over the past few years, my job has become more and more demanding”* as part of the effort subscale.

The prospects index focuses on job insecurity, employment status, career prospects, and downsizing. According to the EWCS [23], 16% of workers in Europe fear losing their job in the upcoming six months. Such job insecurities were described by our participants as stress inducing and were confirmed by a study among university staff [7]. Furthermore, unstable employment status due to non-permanent contracts, lack of positive career prospects, and downsizing by not replacing retiring staff were highly stressful for our participants. The full version of the Job Content Questionnaire which was designed to operationalize the Job Demand-Control model [18], includes a job security subscale with items such as *“During the past year, how often were you in a situation where you faced job loss or layoff?”*. The Effort-Reward Imbalance model [19] also includes the issue with items such as *“My job promotion prospects are poor”*, belonging to the rewards subscale.

Two of the EWCS job quality indices [23] were not discussed by our participants. First, the participants did not refer to the physical environment index, which assesses the exposure to physical risks at the workplace, such as noise, chemicals, and infectious products. Most likely, this was not mentioned due to the office environments where the participants work, where there are little to no physical work demands. Second, the participants did not mention the earnings index, which measures the monthly income of workers. Perhaps this was not mentioned because it was considered to be more of an underlying worry than a common stressor.

Some of our categories seemed not to be covered by the EWCS [23]. We therefore developed organisational and physical status as two further indices. The organisational index includes structural and process-oriented stressors such as setting task priorities in a team and financial stressors such as waiting for and worrying about reimbursements of work expenses. The physical status index assesses stressors related to employees’ health such as problems with eyesight or diminished sleep quality. Neither the Job Demand-Control model [18] nor the Effort-Reward Imbalance model [19] include these additional indices in their models or corresponding questionnaires. A possible explanation for these omissions is that it could be a consequence of the conceptual framework that is commonly used in research on the psychosocial work environment and health.

According to Rugulies and Martikainen [38,39], the framework is based on four levels of sources for psycho-physiological changes and health-related behaviours, which in combination may influence workers’ health. The four levels include (I) macro-level economic, social, and political structures; (II) meso-level workplace structures; (III) meso-level psychosocial working conditions; and (IV) individual-level experience and cognitive and emotional processes [38,39]. While the EWCS job quality indices [23] and hence the results that we structured within the framework can mostly be categorised as meso-level psychosocial working conditions, our two additional indices belong to other levels. 

Our organisational index belongs to meso-level workplace structures. While some stressors were mentioned which fit into other categories (such as deadlines), our participants stressed the organisational aspects of these. They viewed their frustration as a direct consequence of poor work organisation. Previous research has shown that a negative work climate in the organisation can lead to the underreporting of stress-related issues due to fear of it having a negative impact on one’s work relationships. Furthermore, structural issues at work can negatively affect workers’ willingness to discuss work-related stress, potentially leading to bullying or burnout [40]. Another stressor in line with our results is organisation-imposed work overload due to the lack of individually altering responsibilities and duties based on personal needs and preferences. Compared to self-initiated work overload, organization-imposed work overload proved to be associated with worse job satisfaction, higher emotional exhaustion, and higher risk of work-life conflict [41]. 

Our physical status index is categorised as a work stressor and not a stress outcome. While research generally considers it as a stress outcome [3], physical status was specifically mentioned as a stress risk factor by our participants. Previous studies confirm that adverse physical health can lead to work stress. Reoccurring headaches are correlated to lower quality of life, lower self-rated work ability, depressive symptoms, and work stress [42]. Additionally, sleep disturbances were found to predict higher perceived work demands and work stress, as well as a lower degree of perceived support and control at work [43].

Hence, while the EWCS [23] and the models [18,19] focus primarily on meso-level psychosocial working conditions, our results from the focus groups show that our participants reported additional stressors from other levels, influencing their work stress experience. This demonstrates certain limitations of the EWCS [23] and widely used theories such as the Job Demand-Control model [18] and the Effort-Reward Imbalance model [19].

### Strengths and Limitations

The main strength of this study is the added value of a qualitative approach in occupational stress research. Specifically due to the inductive phenomenological approach, results beyond existing frameworks were found. Some limitations should be mentioned. First, in Belgium, seven institutions were contacted, four of which did not participate due to limited time resources. This potentially introduced selection bias. Second, data saturation remains debatable due to the diversity of occupations across our participants. However, we believe that our results reflect a wide spectrum of work stressors experienced by office workers. Third, the EWCS [23] is not solely based on office workers but includes workers from different sectors. Nevertheless, it proved to be suitable for structuring our results. Fourth, this study is based on qualitative research, which does not allow generalisability of the results. However, our findings can be of major importance for the specialists in this research field.

## 5. Conclusions

Our results confirm data from existing occupational stress literature as well as widely used and well-known job-quality frameworks and models. The topics of work intensity and social environment have proven to be especially relevant. The EWCS [23] was used as a theoretical framework to structure and present our results. However, two out of the seven EWCS job quality indices [23] were not discussed by our focus group participants, namely, physical environment and earnings. A relevant addition to the research field is that we found results going beyond the EWCS [23], the Job Demand-Control model [18], and the Effort-Reward Imbalance model [19] by exploring structural and process-oriented stressors, financial stressors, and stressors related to employees’ health. Our focus group study thereby confirms the multi-level complexity of work stress experiences of office workers, including a broader spectrum than the meso-level psychosocial working conditions. This complexity should be taken into account in further research and occupational stress prevention efforts.

## Figures and Tables

**Table 1:** Overview of the job quality indices and their indicators based on the EWCS [23] including exemplar quotes. An asterisk (*) signifies indices and indicators added based on the FGs.

Job-Quality Indices	Indicators	Exemplar Quotes
Physical environment	Posture-related (ergonomic)	
	Ambient (vibration, noise, and temperature)	
	Biological and chemical	
Work intensity	Quantitative demands	*“Yes, I would say time pressure, well, but not in the sense, I don’t know, that I couldn’t schedule it properly, but the thing, I don’t know, the tasks just keep coming additionally, right, additional tasks, right. So yes, too extensive workload.” (FG6)*
	Pace determinants and interdependency	*“And that the client see that as things always that you are only working on his project and that they don’t realize that you don’t have only one project, you have multiple. And they think you are, they are the only one who you have to think about, which is not true of course. Eh, but it’s also what the client, I think it’s also part of what the client expects. Because they, they go to this big office and they see a capacity so it should be fine. You cannot really tell them or explain that to them because they’ll not understand that is. But you have so many people, how can that be?! That’s … that’s indeed it’s a stressful situation.” (FG3)*
	Emotional demands	*“So I have no [laughs], … not many friendly contact.” (FG2)*
Working time quality	Duration	*“… but then there was traffic … [laughs] so was a little stress.” (FG2)*
	Atypical working time	
	Working time arrangements	
	Flexibility	*“The work*-*life balance is for me very difficult … I want still to do something for work because then it’s finished but if I do that then I feel regret that I don’t take private time but if I do my private time then I regret that I was not working.” (FG1)*
Social environment	Adverse social behaviour	*“Lots of things could have been avoided by different communication … it’s the way how you communicate with people. If it’s an email, if it’s a very different approach when you just have somebody in a meeting and explaining to them … that’s a very different approach which creates stress or tension that’s not really necessary. So that’s a bit a big thing just to have open communication about other things instead of just doing it through email or… keeping it to yourself.” (FG3)*
	Social support	*“Then you experienced that she, eh, doesn’t really want to do that and she actually doesn’t do it, not at all. So after three weeks I think I had some, five adjusted problems. And, yeah, each three, two or three days when I was at the office and I checked, she really didn’t want to do it. And, eh, after three weeks, we decided that, yeah, she really didn’t want to do anything for me… so, that was really strange, it was the first time that actually someone came in and that, yeah, didn’t really want to help and didn’t want to cooperate in, in the team. That was really strange.” (FG3)*
	Management quality	*“It is, let’s say, something to work on for the management here. Because they, it’s, I, I only once have experienced that somebody said to me ‘you have done a good work.’” (FG3)*
Skills and discretion	Cognitive dimension	*“Quite often you have a schedule in your mind like, ok, by then I need to do that, by then that and quite often with all of this just pop up like, yeah, can you still do that and quite big challenges not just small ones. So it’s often quite difficult to manage deadlines because there are a lot of unforeseen challenges.” (FG1)*
	Decision latitude	*“We have a lot of liberty … freedom in our job and it’s very important… for me important. But I know that this can give stress for people who just don’t have the limits and can go a little bit in all the ways they want. So that’s maybe something happens a lot here.” (FG2)*
	Organisational participation	*“We feel that if something were to change here and if there would be more dialogue, that is, perhaps … what is wrong, more dialogue with the people who work in individual areas, because they are the ones with the best solutions, let’s say, right. And it happens often that they are instead thinking about these solutions in a top-down manner, instead of thinking from the point where people know, in this case they would simply get to solutions faster, right. And that’s for every area and that is then, hmm… that is, for, for those people, that aren’t us perhaps … we do talk quite a bit … but some people know how they could organize things, but they cannot speak out at all because, well, they are simply afraid.” (FG5)*
	Training	*“What I could say is one thing that I could say stressful is that the person who’s supposed to give the handover and to coach me, train me, is sick for the moment. So not really easy to start that way so I need to pick up information where I can.” (FG2)*
Prospects	Employment status	*“So not everyone have long-term contracts. They have short contracts and sometimes we don’t have the answer enough on time. And so I think it’s really stressful for the people because they don’t know if they will stay or not. I had experienced to someone who didn’t know one week before the end of his contract. That’s something, sometimes we cannot do or we don’t know how to change because, yeah, it’s budget and organization.” (FG2)*
	Career prospect	*“Applying for project and having a lot of negative, failure projects. So something you submit and you get it back, they don’t accept.” (FG1)*
	Job security	*“What I also see a lot is that with the restructuration. And the information, they hear something but they don’t know what really gonna happen and the people on top they can’t tell, they have to lie sometimes. It gives a lot of stress to the people because ‘Next week, am I gonna work here? Is this shop gonna be open? Are we gonna move to there?’ And the people on top don’t know yet. And that gives everywhere you feel the same stress because of the uncertainty they heard already.” (FG2)*
	Downsizing	*“The main problem in our department is the lack of staff for the number of project that there are… when I started to work here 14.5 years before, I think that were 5 people who have been retired or out of contract who haven’t been replaced. And the staff was about 55, maximum 60 persons and now we are almost doubled with a lot less [admin] staff.” (FG1)*
Earnings		
Organisational *	Structural and process-oriented *	*“The way I see this, what makes stress for me, like she mentioned, and then for example I am forced to go over this thing again and then I feel like stress, because I feel I need to do a thing again that doesn’t have a goal.” (FG4)*
	Financial *	*“There is also the money issue. Because they have to get the money back for everything they spend and it sometimes, it takes time.” (FG2)*
Physical status *	Employees’ health *	*“And if you have a health condition, currently for half a year, my eyes are playing with me, I cannot read in the evenings, I cannot read over the weekends as much as I used to and that means … enormous quantity of books that lay there and I haven’t read them. And no one will instead of me.” (FG5)*

## Data Availability

The data presented in this study are available on request from the corresponding author. The data are not publicly available due to privacy-related reasons.

## Appendix A

**Table A1:** Predefined Semi-Structured Focus Group Guide.

**Introduction and purpose (approximately 10 min)** Introduce yourself as the moderator (your professional role and academic background—responsible for asking the questions and guiding the discussion) and let your second researcher introduce himself/herself (his/her professional role and academic background—responsible for taking notes—preferably handwritten since it is considered as less disturbing, the audio recording, the time management, and double checking at the end if all questions have been discussed) Explain the aim of the focus group: “To understand feelings, meanings, interpretations, opinions, and perceptions of a selected group of people to gain an understanding of a specific issue from the perspective of the participants” “We would like to give each one of you a voice, we do not aim to reach consensus and there are no wrong answers” “We chose focus groups to stimulate a discussion among colleagues and to get different perspectives on the topic of stress at work” “We hope to give an incentive for self-reflection on your own work stressors and to stimulate an active intercommunication about stress at work beyond this focus group” Explain what will be done with the results: “This focus group is a preparatory study for our project. We aim to gain profound insights into the sources of work-related stress among office workers” Explain the expectations towards the participants: “You can tell us about your own experiences and feelings, you do not need to feel obliged to report for others. Your opinion counts and your contribution is very valuable to us” “We aim to create an honest und trustful discussion. Therefore, we count on your collaboration to achieve confidentiality of the statements given in this focus group” Our communication rules: We try to ask everyone to say his/her name every time a participant says something (facilitates the transcription afterwards and gives a structure) If it does not work we let the participants take over the discussion and let them speak freely -> take notes of the first words every time someone new is talking or stop the time and write down who is starting to talk at what minute and second “Are there any question so far?” “Can you please introduce yourself and your professional role?” -> do this in a circle **Discussion (approximately 60 min)** Introductory questions: “What is it like to work here?” Linking questions: “What are your first thoughts about stress at work?” “What does stress at work mean to you?” Key questions: “In your experience, what causes stress at work?” “Which stressful situations do you experience the most?” “Which of these causes of stress feels to be the most important one?” “Which stressful situations can you observe among your colleagues?” “If you were asked to advise your employer on what should be done first about stress at work, what would you recommend?” Back-up questions: “Try to remember the last time you felt stressed at work: which task were you focusing on?” (to stimulate the discussion) “We have heard different causes of stress already. What is your opinion about it?” (giving the word to a rather quiet participant) “We really appreciate your inputs. Would it be alright if I pass the word to another participant for now?” (directing towards a rather active participant) **Conclusions (approximately 10 min)** Paraphrasing questions: “How well does that capture what was said?” “Have I managed to adequately summarize the discussion?” Closing questions: “Is there anything we have missed?” “Is there something you would like to add?”
